# Statistical Models for Vaginal Microflora: Identifying Women at Risk for Group B *Streptococcus* Colonization as a Test of Concept

**DOI:** 10.1155/S1064744997000586

**Published:** 1997

**Authors:** Mei-Ling T. Lee, Robin A. Ross, Andrew B. Onderdonk

**Affiliations:** 1 Channing Laboratory Departments of Medicine and Pathology Brigham and Women's Hospital and Harvard Medical School Boston MA USA; 2 Channing Laboratory Harvard Medical School 181 Longwood Avenue Boston MA 02115 USA

## Abstract

*Objective:* The purpose of this study was to formulate a statistical model that relates human microflora to probabilities for vaginal colonization by group B *Streptococcus* (GBS).

*Methods:* Longitudinal observations of total bacterial concentrations at various times during the menstrual cycle were obtained from overtly healthy, non-pregnant, menarcheal women. During each menstrual period and at appropriate intermenstrual times, the duplicate swab technique was used to sample the vaginal vault to obtain microbiologic samples. Women were identified as being colonized with GBS if their samples contained faculative gram-positive cocci. The method of generalized estimating equation (GEE) was used to model the longitudinal data set.

*Results:* Concentrations of *Corynebacterium* sp., *Streptococcus* spp., and total anaerobic bacteria were found to be risk factors for GBS colonization. The sensitivity of the predictive model is 84% and the specificity is 79%.

*Conclusions:* Although vaginal cultures for GBS are routinely performed to detect colonization, the statistical model described identifies associated risk factors which may be important determinants for GBS colonization.


Infectious Diseases in Obstetrics and Gynecology 5:336–340 (1997)
© 1998 Wiley-Liss, Inc.

### Statistical Models for Vaginal Microflora: Identifying Women at Risk for Group B *Streptococcus* Colonization as a Test of Concept

**Mei-Ling T. Lee,* Robin A. Ross, and Andrew B. Onderdonk**
*Channing Laboratory, Departments of Medicine and Pathology, Brigham and Women's Hospital and Harvard Medical School, Boston, MA*

ABSTRACT

***Objective:*** **The purpose of this study was to formulate a statistical model that relates human microflora to probabilities for vaginal colonization by group B *Streptococcus* (GBS).**

***Methods:*** **Longitudinal observations of total bacterial concentrations at various times during the menstrual cycle were obtained from overtly healthy, non-pregnant, menarcheal women. During each menstrual period and at appropriate intermenstrual times, the duplicate swab technique was used to sample the vaginal vault to obtain microbiologic samples. Women were identified as being colonized with GBS if their samples contained faculative gram-positive cocci. The method of generalized estimating equation (GEE) was used to model the longitudinal data set.**

***Results:*** **Concentrations of *Corynebacterium* sp., *Streptococcus* spp., and total anaerobic bacteria were found to be risk factors for GBS colonization. The sensitivity of the predictive model is 84% and the specificity is 79%.**

***Conclusions:*** **Although vaginal cultures for GBS are routinely performed to detect colonization, the statistical model described identifies associated risk factors which may be important determinants for GBS colonization. Infect. Dis. Obstet. Gynecol. 5:336–340, 1997.** © 1998 Wiley-Liss, Inc.

KEY WORDS
*Corynebacterium* sp.; *Streptococcus* spp.; total anaerobic bacteria; vaginal ecosystem; predictive statistical modeling

Group B *Streptococcus* (GBS) is a gram-positive organism that forms diplococci or chains, groups within Lancefield group B, and produces a polysaccharide capsule. Antigenic variation of the capsular polysaccharide forms the basis for the serotyping system within group B. GBS are considered normal vaginal microflora under most circumstances. The role of the normal microflora for both health and disease has received increased attention in recent years as the impact of various interventions on the microflora has been identified. Microbiologic surveillance of human ecosystems often includes qualitative and, on occasion, quantitative bacteriologic culture for organisms of interest. Such methods are most often akinetic and do not identify relationships among microorganisms which may be important to the colonization of a particular environment. For diseases caused by members of the normal microflora, understanding such relationships may be significant in preventing both colonization and subsequent infection. During the past several years, we have developed statistical methods that help identify possible relationships between various members of the normal microflora. Several relationships identified in this manner have subsequently been elucidated in greater detail.

Contract grant sponsors: Tambrands, Inc., and SmithKline Beecham Consumer Products.
*Correspondence to: Dr. Mei-Ling T. Lee, Channing Laboratory, Harvard Medical School, 181 Longwood Avenue, Boston, MA 02115.

Received 18 March 1997

Accepted 31 October 1997

The real test of these methods is, however, application of the predictive model to an actual in vivo situation.

Clinicians focused attention on Lancefield GBS during the 1970s. GBS is a major cause of neonatal sepsis and meningitis in the United States with an incidence rate of 18/10,000 live births and a mortality rate of 6%.[1,2] GBS, or *Streptococcus agalactiae*, can be isolated from cultures of the rectum, vagina, cervix, urethra, skin, and pharynx.[1] As is the case for most streptococci, GBS is considered normal microflora, even though it can be an invasive pathogen in a variety of clinical settings. For neonates, however, exposure to GBS during birth can lead to development of disease. Most strategies for decreasing the risk of neonatal GBS disease involve the use of antibiotic prophylaxis. This method requires prenatal screenings for detection of GBS, which is problematic due to transient or intermittent colonization by this organism, which may be missed by ordinary culture methods.

In this study, the GBS colonization status of healthy, asymptomatic women was used as a way to test predictive modeling methods using a statistical model developed to identify women at risk for carriage of GBS. A statistical model was fitted to an in vivo data set describing the vaginal ecosystem. The model defines a statistical relationship among microorganisms which allows for the identification of women who have an increased relative risk of carriage of GBS.

### MATERIALS AND METHODS

### Data Set

Over the past 10 years a large data set containing both quantitative and qualitative microbiologic information was assembled from in vivo studies describing the healthy vaginal environment.[3–5] This data set includes information about total bacterial concentrations, as well as concentrations of the dominant isolates at various times during the menstrual cycle. Non-pregnant, menarcheal women volunteered to be monitored through successive menstrual cycles. Pelvic examinations were performed on each woman prior to the start of the protocol, and samples for culture of *Chlamydia trachomatis* and *Trichomonas vaginalis* were obtained. Criteria for exclusion included pregnancy, genital abnormalities, use of contraceptive spermicides, vaginal infections including bacterial vaginosis detected clinically, abnormal Papanicolaou test results, hysterectomy, antimicrobial therapy, douching 1 month or less before the start of sampling, positive tests for human immunodeficiency virus (HIV) or hepatitis A or B virus antibody, or a history of pelvic inflammatory disease or ectopic pregnancy. The data set is biased toward normal healthy vaginal microflora, a desired result of the exclusion criteria. In addition, the data set includes women using different catamenial products and in different stages of the menstrual cycle. Both of these variables have been previously shown not to significantly alter the vaginal microflora.[4,5,9]

### Microbiologic Cultures

During each menstrual period and at appropriate intermenstrual times, the duplicate swab technique was used to sample the vaginal vault to obtain microbiologic samples. Serial decimal dilutions of the sample were plated onto various selective and non-selective media for the recovery of facultative and obligately anaerobic microorganisms. For the recovery of anaerobic bacteria, the following media were used: prereduced Brucella base agar with 5% sheep blood containing hemin and vitamin K1, each at 10 mg/l (BMB); BMB with 150 mg neomycin sulfate/l; and prereduced Brucella base agar with 5% laked sheep blood, 100 mg kanamycin/l, 7.5 mg vancomycin/l, and hemin and vitamin K1, each at 10 mg/l. The media used for the recovery of facultative organisms were 5% sheep blood in tryptic soy agar, mannitol salt agar, Chocolate agar, and MacConkey agar. All colony types were isolated and identified by established criteria as described previously.[3–6] Women were identified as being colonized with GBS if their samples contained facultative gram-positive cocci that were catalase negative, did not grow on bile esculin azide agar medium, and produced the CAMP factor. Verification of GBS isolation was performed using long chain fatty acid analysis and the MIDI system (Microbial Identification System, Microbial ID, Inc., Newark, DE). Repeated longitudinal measurements of the concentration of members of the vaginal microflora including *Lactobacillus* spp., *Corynebacterium* sp., *Streptococcus* spp., *Staphylococcus* spp., *Escherichia coli*, *Prevotella bivia*, *Gardnerella vaginalis*, total anaerobic bacteria counts, total facultative bacteria counts, and counts for over 80 additional species routinely isolated as part of the vaginal microflora were obtained. Bacterial concentrations were expressed as $\log_{10}$ colony forming units (cfu) per gram of sample.

### Statistical Model

As a preliminary analysis to identify variables predictive for GBS colonization, a correlation matrix was used to identify variables which were highly correlated with the concentration for any *Streptococcus* spp. (STREP). The field within the data set for *Streptococcus* spp. included a variety of streptococcal groups, including the subset of counts for GBS, all of which could be separated from each other during analysis.

Observations with missing information on *Corynebacterium* sp., *Streptococcus* spp. and total anaerobes were excluded from the analysis. Accordingly, the data set used in the model is comprised of 536 repeated measurements taken from 58 women. The data are not composed of uniquely independent samples, and conventional logistic regression methods cannot be applied to the data. Therefore, we used the method for a generalized estimating equation (GEE), which generalizes the logistic regression by taking into account the within-person dependence between sampling[7] as part of the analysis (SAS macro software, Johns Hopkins University, Baltimore, MD).

For each woman, let Y(t) denote the dichotomous response variable for GBS colonization at the $t^{th}$ visit. Y(t) = 1 indicates that a woman has GBS colonization at the $t^{th}$ visit, and Y(t) = 0 indicates a negative result. Corresponding to the outcome variable Y(t) is a set of independent variables $X_1(t)$, $X_2(t), \ldots, X_k(t)$ containing different bacterial concentrations which are used as risk factors in the equation to predict the occurrence of GBS. Let P(t) denote the probability that Y(t) = 1 at the $t^{th}$ visit. Define the logit transformation as

$$\text{logit } P(t) = \ln\,[P(t)/(1 - P(t))].$$

If we assume that the logit of the probability that a women is colonized by GBS on the $t^{th}$ visit is a linear function of the various bacterial concentrations obtained during sampling, then the GEE regression model takes the form

$$\text{logit } P(t) = B_0 + B_1 * X_1(t) + B_2 * X_2(t) + \ldots + B_k * X_k(t) + e$$

**TABLE 1. Summary statistics of bacterial concentrations for the covariate variables**

| Variable | N | Mean[a] | SD[b] |
|---|---|---|---|
| | *Non-GBS Observations: Y[t] = 0* | | |
| STREP | 419 | 4.462 | 1.890 |
| CORYNE | 419 | 6.121 | 1.860 |
| TOTALAN | 419 | 8.357 | 1.083 |
| | *GBS Observations: Y[t] = 1* | | |
| STREP | 117 | 7.533 | 1.497 |
| CORYNE | 117 | 5.554 | 2.198 |
| TOTALAN | 117 | 8.323 | 1.202 |

[a]Bacterial concentrations were expressed as log colony forming units per gram of sample.
[b]SD = standard deviation.

where e denotes random sampling error. Stepwise forward regressions of the GEE models were then conducted to evaluate possible risk factors.

### RESULTS

### Regression Model

The results of correlation analysis indicated that *Corynebacterium* sp. (CORYNE) has a correlation −0.27 ($P$ = 0.0001) with *Streptococcus* spp. Although not significantly correlated with *Streptococcus* spp., the total count for obligate anaerobes (TOTALAN) had a correlation of 0.137 ($P$ = 0.001) with *Corynebacterium* sp., suggesting a linkage between these two risk factors. The statistics for the bacterial counts of these variables are summarized in Table 1.

Of the 536 observations included in this study, 117 (22%) were identified by culture as GBS positive. These culture results were used as the outcome variable in the analysis. Applying the GEE method with interchangeable correlation for between-visits association and a stepwise regression method, we obtain the following model for the data set:

$$\text{logit } (P(t)) = -4.653 + 0.713 \text{ STREP} + 0.054 \text{ CORYNE} - 0.156 \text{ TOTALAN}.$$

With the above model, a threshold value Pc as the cutoff probability can be selected for predicting GBS colonization.

A predictive decision rule was made as follows: A woman is predicted to be colonized by GBS if her predictive probability value, P(t), at the $t^{th}$ visit is greater than the threshold value, otherwise she is not predicted to be colonized by GBS. We consider the threshold value Pc = 0.24, which is greater than the observed probability (0.22) of GBS colonization.

As a measure of accuracy of the GEE model for predicting GBS colonization we computed the sensitivity and specificity for the model. Sensitivity is the proportion of GBS culture positive observations that the GEE model correctly predicts to be GBS positive. For the threshold value Pc = 0.24, 84% of the 117 GBS culture positive observations contained in the data set were successfully predicted by the model as having GBS colonization. Specificity is a measure of accuracy for predicting non-GBS observations. It is the proportion of GBS negative observations that the GEE model predicts to be GBS negative. Of the 419 GBS culture negative observations, 79% of them were predicted by the model as not having GBS colonization.

### DISCUSSION

The bacterial populations residing within the human vaginal vault are members of a complex ecosystem. The mechanisms that control bacterial populations in this environment are as yet to be understood. The concept of statistical modeling applied to an ecosystem provides a new tool for microbiological analysis.[8,9] The model described in this research is important in that it defines a statistical relationship among the complex interactions of the various microbial species that make up the vaginal microflora such that a linear predictive equation is obtained for identifying possible GBS cases. Of the many different microbial species present among the vaginal microflora, the obtained model is able to give a reasonable prediction on the occurrence of GBS based upon the *Streptococcus* spp., *Corynebacterium* sp., and total anaerobic bacteria counts. The predictive accuracy for this statistical model is likely understated because the microbiologic cultures used to assemble the data base were not designed to specifically detect GBS and it is possible that low levels of colonization were not detected. Although it is easier to simply culture a given subject for GBS than to perform quantitative cultures for modeling purposes, the relationships between microbial populations cannot be evaluated from such clinical studies. Because the model resulting from this research is in a simple form with only three risk factors (CORYNE, STREP, and TOTALAN), it could be easily adapted for clinical studies to identify women prone to GBS colonization, even if GBS is not isolated. Indeed, the microbiologic data used to establish this model were not set up to specifically isolate GBS, but rather to identify the dominant vaginal microbiologic components. Moreover, the three identified factors for predicting GBS may be important for future study and evaluation.

The analysis of sensitivity and specificity of the model demonstrates the goodness-of-fit of the model by comparing observed GBS cases to predicted results according to a range of cutoff probability threshold values. In addition, using the coefficients of the obtained model, we can approximate the relative risk of a woman having GBS infection with respect to the increases or decreases of the bacterial counts for STREP, CORYNE, or TOTALAN. Although the data presented represent a single application, the statistical model may provide a technique for future preventive epidemiological studies. Understanding the relationship between microbial populations may aid in understanding vaginal colonization risk factors for women.

We believe that the application of statistical modeling strategies, such as those described, represents an important new approach to understanding the underlying mechanisms responsible for microbial colonization. While GBS is only a convenient example of this strategy, it may be possible to apply similar methods to a variety of other components of the vaginal microflora. Such studies are ongoing at the present time.

### REFERENCES

1. Schuchat A, Wenger JD: Epidemiology of group B streptococcal disease: Risk factors, prevention strategies and vaccine development. Epidemiol Rev 16:374–402, 1994.
2. Anthony BF, Okada DM, Hobel CJ: Epidemiology of group B streptococcus: Longitudinal observations during pregnancy. J Infect Dis 137:524–530, 1978.
3. Onderdonk AB, Polk BF, Moon NE, Goren B, Bartlett JG: Methods for quantitative vaginal flora studies. Am J Obstet Gynecol 122:777–781, 1977.
4. Onderdonk AB, Zamarchi GB, Rodriguez ML, Hirsch ML, Munoz A, Kass EH: Qualitative assessment of vaginal microflora during use of tampons of various compositions. Appl Environ Microbiol 53:2774–2778, 1987.
5. Onderdonk AB, Zamarchi GB, Rodriguez ML, Hirsch ML, Munoz A, Kass EH: Quantitative assessment of vaginal microflora during use of tampons of various compositions. Appl Environ Microbiol 53:2779–2784, 1987.

6. Onderdonk AB, Delaney ML, Hinkson PL, Dubois AM: Quantitative and qualitative effects of douche preparations on vaginal microflora. Obstet Gynecol 80: 333–338, 1992.
7. Liang KY, Zeger SL. Longitudinal data analysis using generalized linear models. Biometrika 73:13–22, 1986.
8. Ross RA, Lee MLT, Delaney ML, Onderdonk AB: Mixed-effect models for predicting microbial interactions in the vaginal ecosystem. J Clin Microbiol 32:871–875, 1994.
9. Lee MLT, Ross RA, Delaney ML, Onderdonk AB: Predicting abnormal microbial population levels in the vaginal ecosystem. Microbial Ecol Health Dis 7:235–240, 1994.
